# Supplementary material for: Patterns of Intron Gain and Loss in Fungi
Source: PLoS Biol. 2004 Nov 30;2(12):e422. doi: 10.1371/journal.pbio.0020422 (PMC532390; doi:10.1371/journal.pbio.0020422)
Supplement: Table S1 — Also available at http://genes.mit.edu/NielsenEtAl/. (4.3 MB ZIP). [file pbio.0020422.st001.zip › NielsenEtAl/html/1377.html]

AN4486.1.NCU02094.1.MG00672.1.FG00719.1


```
 CLUSTAL W (1.82) Multiple Sequence Alignments - Introns Inserted


Sequence 1: NCU02094.1	734 aa
Sequence 2: MG00672.1	740 aa
Sequence 3: FG00719.1	743 aa
Sequence 4: AN4486.1	731 aa
Alignment Length: 784 aa
Number Identitical Residues: 259 aa
Alignment Score (without introns) 14255


MG00672.1 	MDHGAAASLSPTSPVSARSHVGANGGPKRKRSVGGLASTIDSSPGSPGLDDDHGDHHEKK
NCU02094.1	ME-------SPEFSAGMRNQGPNGGPPKRKRSIMESSPASTGDNDREERDDGSPETKGGR
FG00719.1 	MAASGAASMSPTSPVSSR-HGHAHGNSNGIKRKRSSVAGIDSSPGS-GLDDHDAEQ-EKK
AN4486.1  	--------MGGIGHTSISPAVASDGAGQARKRNAG-MAGLDSSPGSIDDVEEVENRDEKR
          	         .    ..        *  :  :      .   .. .     :   :    :

MG00672.1 	RQPGVKRACNECRQQK0LRCDVVQDPFQGCTRCGRLKLECKIESNFKRVGKRSKHAEMEK
NCU02094.1	RLPGVKRACNECRQQK0LRCDVVQEPFSSCSRCNRLHLECKIESNFKRVGKRSKHAEMEK
FG00719.1 	RQPGVKRACNECRQQK0LRCDVVQDPFQSCSRCNRLKLECKIESNFKRVGKRSKHAEMEK
AN4486.1  	RQP-VKRACNECRQQK0LRCDVVQDPWTDCSRCRRLKLECKIESNFKRVGKRSRNAEMER
          	* * ************ *******:*: .*:** **:****************::****:

MG00672.1 	EIDMLRRAVQRARTAGFIDEDD--------SMHSQLQSPIVNNVYSHTRNPS-----LMG
NCU02094.1	EIERLRRAVQSAKSQGFVVDEDDDNNTPGNATHSQLHSPVINSHYSHTRNPS-----LMG
FG00719.1 	EIDRLRRNLARAKSQGFTPEGADD------DMDSPAAASNPTYTHTHTRNPS-----LMG
AN4486.1  	EIIELRKQIASVQSGSVGQQQSHLPSAQQTPKQENPSNQVSPARAYQTPSAMSAADQFMG
          	**  **: :  .:: ..  :     .:  .  ..            :* .. :::..:**

MG00672.1 	SEEAVSSLLHLKRG-------YTIPRIVRSLDEVQLTEDAEAQLFDI~FFRHYHPTLPFL
NCU02094.1	SDEAVSSLLHLKRGGS-----YAMPRIVRELEDFRITEDDEGVLFND~YFHYYHPFLPFL
FG00719.1 	SDEAVSSLLHLKRGGN-----YNMPRYSHELENVQLTEDDVNNLFSQ~FFQFYHPMLPFL
AN4486.1  	SHEAVASLLDLRSGYDGSSYMRNGNQHFKRIEDVAVVPERVTELFDL2FFTYYHPFLPFL
          	*.***:***.*: * ..::      :  : :::. :. :    **.  :* .*** ****

MG00672.1 	NPQMTPEAYYQQHTLLYWVIVAIASRRYP--ADPTLLGNISRPLTQLLWSTIGEVPGNYC
NCU02094.1	NPHQTPDQYYQQYPLLYWAIVAVASRRYG--HDRSFLTKLSSPLTRLLWTTIGEVPQSYF
FG00719.1 	NPRQAPDLFYQQHPLLFWSIVSVAARRFSPPNVPALLSNLSGPLTRFLWSTIGEVPSNYY
AN4486.1  	DREQSPEEYYTVSPLLFWTIISVGARRYQ--PDPNLLNSLAGPVTRLVWSTLADVPQSYH
          	: . :*: :*   .**:* *:::.:**:       :* .:: *:*:::*:*:.:** .* 

MG00672.1 	IVKALCLLCTWPLPTSTSSADPTHILCGVMMKIATGIGLHRPMHINDFSRVQIELNMEQL
NCU02094.1	AVKALCLLCTWPLPTSTSSSDPTHILGGVLMKTATGLGLHRPNHMNDFSRVPIELNKEGL
FG00719.1 	VVKAMCLLCTWPLPTSTTSSDPTHILCGVMMKTATGIGLHRPNNIQDFSRVSVELNKEQL
AN4486.1  	VVKALCLLCSWPFPTSSTSTDPTFMLCGLMIQIAMQLGLHRPSHTQDFSKYRVELVEEEL
          	 ***:****:**:***::*:***.:* *:::: *  :***** : :***:  :**  * *

MG00672.1 	HDRVTTWAVCNIVAQT~IGTGYGQPASTLYDWTLAVRPGDNGPFQLSAELEARLQIERFC
NCU02094.1	HDRIKTWAVANIVAQA~VGTGYGQPATTLYDWTLATRQGDTSPASLGPELEARLQIERFC
FG00719.1 	SDRVTTWAVCNIVAQS~VGTGYGQPASTLYDWTLASKLGEESAFQLSRELEARLQIERFC
AN4486.1  	RDKVRTWATCNIVAQR2VATGYGQPPCTLYDWTLSSQESMDPNFKLPESIRHRLDIEKFC
          	 *:: ***..*****  :.******. *******: : .     .*  .:. **:**:**

MG00672.1 	DKVSKEMYSNASDPRGVAGDEHRSILMRVFRREYSELNANILSRDLSP~VVNLHLKAAGL
NCU02094.1	DKVSKEMYSNASDPRGVAGDEHRAMLMRVYRRDYNELAASVLSKGVSP~VVNLHLKAAGL
FG00719.1 	DKVSKEMYSNASDPRGVAGDEHRAMLMRVYRRDYAELHASVLSRELGP~FVNLHLRAAAL
AN4486.1  	DKVTKALYTNRRDPVGLCGSQERSTLTSLLSRDLDELETQLKPHN-DY1ITDLYLRASNL
          	***:* :*:*  ** *:.*.:.*: *  :  *:  ** :.: .:  .  ..:*:*:*: *

MG00672.1 	HLRLAAFFDSSKTQTYVDDLKELFRATTSFLDAVLEGD---YDTNPQEQGGNDTLKYATN
NCU02094.1	HLRLAGFFDSNNSPNYLDDLMGLWRATTSFLDFILDGDPSCHDHYSYQYRDQYMLKYASN
FG00719.1 	HMRLAGFFDSSKTPGYMDDLMGLWRATTNFLDEVLQAD---KDTTPGDGGGN-LLLHCSN
AN4486.1  	HLHLSAFFDDFTAKDYRERLLSLFFAATTFLEAAMKLQ---TQVGP-------MLSNTPY
          	*::*:.***. .:  * : *  *: *:*.**:  :. :    :  .        *   . 

MG00672.1 	YIQQMLVATGFVLLKLMRSFFAKIIDFERGRLLFHRTSQAIRATSVNRNDLSWRLAELMV
NCU02094.1	FIQQMLVAAGFSLLKLLRSFFAKIIDFDRGRELFHRTIQAIRTTSVIQNDLYWRLAELMV
FG00719.1 	YIGQMMVAAGFALLKLMRSFFSKTIDFQRGRSLFHQTIRAIRTTSVVHNDLQWRLAELMV
AN4486.1  	YIYQMMVAGGCTLLKLCKSFFASHIDMDYAKGLFNRTIWAIRSVSVISNDLPERLAEVLA
          	:* **:** *  **** :***:. **:: .: **::*  ***:.**  ***  ****::.

MG00672.1 	QMWNGA-RVDP-TYMESG-----MIDDSLQLKVRCRHSMSLVFDSIWAWREDYEARGR--
NCU02094.1	QIWNGAGRENPNAYRPDGTDP--VVDDSLQLKVRCRHSMSLVFDSIWHWREEYQARGR--
FG00719.1 	QMWNGARLDNNQHVFNSNDDLPIQMDDSLQLKVRCRHSMSLVFDSVWRWREDFQAIGR--
AN4486.1  	QMWKLGSTPSPKPPPDSV-----EMDDSLMLKVRCRMSMSLLFDSVWRWREDTRTKGRNI
          	*:*: .   . .    .       :**** ****** ****:***:* ***: .: **. 

MG00672.1 	--GSLD~A1LKNPTNPDSANESSASSTHLDTTLMP----PSHGLTASLIEPTPAPANSGG
NCU02094.1	--GTLD~A1LKQPTNPDSANESSASSTHLDSTLAP----PSHGIPN---LGLAAAASNGS
FG00719.1 	--GTID~A1AKQPTNPDSANESSASSTHMDNTLVSSHTLPPSSLLNTNTNNNNIPTTNGA
AN4486.1  	EGDSLT1Y~LKNPTNPDSNADSSASSSVGPPRTSP-------GLATGDPSLAPALLPQSN
          	...::     *:******  :*****:       .       .:             .. 

MG00672.1 	LTPLGASGLSGNSNNGGTQNSAALLTTLGGYDANATAYDFFDPQHWMLDNLVDFGYSFGG
NCU02094.1	ITPGGGS-----TTMGGNQQ----LMGMNGFDT----LDVWDPQHWMLDGLLDFNYTF--
FG00719.1 	LTPGATG----IPAVSAAHN---MMAGVSYGDTTN--FDFFDPQHWMLDGLLDFNYSF--
AN4486.1  	MGVQSGN------NIGSLPSG---FMEPN--------YEVFDPLNWLLDGLVDLPYPYS-
          	:   . .        ..  ..   :   .         :.:** :*:**.*:*: *.:. 

MG00672.1 	VQALET----
NCU02094.1	VPPIEGA---
FG00719.1 	VPALEGA---
AN4486.1  	VSGMEAQGIA
          	*  :*  . :
```
